# Editorial to “Associations of the Fibrosis‐4 index with left atrial low‐voltage areas and arrhythmia recurrence after catheter ablation: Cardio‐hepatic interaction in patients with atrial fibrillation”

**DOI:** 10.1002/joa3.13065

**Published:** 2024-05-14

**Authors:** Satoshi Higa

**Affiliations:** ^1^ Cardiac Electrophysiology and Pacing Laboratory, Division of Cardiovascular Medicine Makiminato Central Hospital Okinawa Japan

**Keywords:** atrial fibrillation, cardio‐hepatic interaction, catheter ablation

In the current issue of the *Journal of Arrhythmia*, Yamada et al.^1^ retrospectively evaluated the association of the fibrosis‐4 (FIB‐4) index with left atrial low‐voltage areas (LA LVAs) and arrhythmia recurrences postcatheter ablation (CA) in patients with atrial fibrillation (AF) (*n* = 343). In this study, patients with FIB‐4 indices ≥1.3 had higher prevalences of LA LVAs (>5 cm^2^) than those without. Furthermore, there was a positive correlation between the quantitative LVA size and FIB‐4 index. In multivariate Cox models, a FIB‐4 indices ≥1.3 were an independent predictor of AF recurrence after a CB‐based PVI without additional LVA ablation. Therefore, the authors proposed a preprocedural assessment of the FIB‐4 index could be a useful predictor of the existence of LA LVAs and AF recurrence after a CB‐based PVI.

The PVs are major sources of triggering foci initiating AF. Therefore, PVI has become the corner stone of AF ablation but still has not been standardized because additional ablation strategies are required to reduce AF recurrence. Previous reports demonstrated that the extent of LVAs revealed by electroanatomic mapping correlated with the progression of atrial electrical remodeling. Thus, additional ablation of the LVAs post‐PVI is one of the key strategies to reduce atrial arrhythmia recurrence. Masuda et al.^2^ evaluated the prognosis of 1488 consecutive patients who underwent AF ablation according to the LVA size. In that study, patients with LVAs were more likely to receive substrate ablation beyond the PVI than those without. Patients with LVAs were more often older and females, patients with a previous history of diabetes mellitus, heart failure, or a stroke. Furthermore, patients with LVAs more often had persistent AF. Masuda et al.^2^ concluded that both an LVA presence and its extent are associated with poor long‐term composite endpoints of death, heart failure, and strokes, irrespective of AF recurrence. Therefore, preprocedural predictors of the existence of LVAs are important for determining the indication for CA, appropriate strategy, and modality.

Liver disease can cause inflammation and autonomic dysfunction, which can contribute to arrhythmogenesis. Huang et al.^3^ reported a high prevalence and incidence of AF in patients with liver disease. Although, liver diseases have been suggested to cause the AF to develop and progress, pathological assessments by liver biopsies are contraindicated in anticoagulated patients. In contrast, the FIB‐4 index is a noninvasive scoring tool that is available for predicting liver impairment and fibrosis by quickly calculating the constitutional and time‐sensitive parameters without an expensive cost. Furthermore, the FIB‐4 index has also been suggested to be a risk assessment tool for several chronic diseases including cardiovascular diseases. The prognostic impact of the FIB‐4 index on risk stratification of cardiovascular events and mortality in AF patients has also been reported. A previous Japanese multicenter study (*n* = 3067) conducted by Saito et al.^4^ evaluated the impact of the FIB‐4 index on the risk stratification of cardiovascular events and mortality in AF patients. This study demonstrated that an FIB‐4 index ≥2.51 was independently associated with cardiovascular events and all‐cause mortality. Furthermore, this study showed that a combined assessment of the FIB‐4 index and CHA2DS2‐VASc score improved the predictive value of cardiovascular events and all‐cause mortality. An FIB‐4 index ≥2.51 was most strongly associated with cardiovascular events and all‐cause mortality in AF patients with high CHADS2 scores. In addition, patients with an FIB‐4 index ≥2.51 had a significantly higher prevalence of both persistent and long‐standing persistent AF with a lower rate of receiving CA procedures. Therefore, patients with an FIB‐4 index ≥2.51 have a more complex AF burdens than those with an FIB‐4 index <2.51. According to this study's results, the FIB‐4 index was an independent prognostic indicator for identifying the AF burden's complexity and also could be a valuable tool for additional risk stratifications in AF patients with high CHADS2 scores.

Recently, Iwawaki et al.^5^ also investigated whether the FIB‐4 index is associated with recurrent AF post‐CA in patients with and without heart failure (HF). Interestingly, there was no significant difference in the recurrence post‐CA among the low (<1.3)‐, intermediate (1.3–2.67)‐, and high (>2.67)‐ FIB‐4 index groups in those with non‐HF. In contrast, the FIB‐4 index was an independent predictor of recurrence in only a specific population, nonparoxysmal AF associated with HF, but not in paroxysmal AF.

The study by Yamada et al.^1^ provided important clinical implications to emphasize the useful predicators of the existence of LA LVAs and AF recurrence post‐PVI, and to determine the appropriate strategy and suitable ablation devices. This is the first observational study to reveal the independent associations between the FIB‐4 Index with LA LVAs and arrhythmia recurrence post‐CB‐based PVI without additional LVA ablation. The limitations of this study are related to the single center, retrospective study design causing a potential bias, should be carefully considered when interpreting the data. The selection of an RF‐ or CB‐based PVI was determined by the operator's judgement. Although, the authors performed multivariate analyses to investigate the association of the FIB‐4 index with LA LAVAs and AF recurrence, a selection bias should be considered. Because of the relatively small population size, the predictive values of the FIB‐4 index by matching all groups regarding age could not be fully evaluated. Voltage mapping of the LVAs was assessed post‐PVI. That is why, it limited evaluating LVAs around the PV antra. The clinical meaning of the existence of patients with small LVAs (<5 cm^2^) should be clarified. Furthermore, the authors did not evaluate the severity of liver fibrosis using abdominal sonography. Therefore, the etiology of the high FIB‐4 index remains unclear.

In aggregate, further investigation of the effects of additional LVAs ablation on the overall outcomes in patients with high FIB‐4 indices is required. Yamada et al.^1^ are to be congratulated for their valuable contribution that highlights the preprocedural assessment of the FIB‐4 index as a predictor of the existence of LA LVAs and the outcomes post‐AF ablation. Their contribution moves the field forward by better understanding of the cardio‐hepatic interaction in patients with AF.

## CONFLICT OF INTEREST STATEMENT

The author has potential conflicts of interest: S.H. is a consultant to Japan Lifeline, Johnson & Johnson, Boston Scientific, and Medtronic, and received speaker's honoraria from Japan Lifeline, Johnson & Johnson, Boston Scientific, Medtronic, Abbott, and Biotronik, and the Boehringer‐Ingelheim, Bristol‐Myers, Bayer, Pfizer, Daiichi‐Sankyo, Mochida and Ono Pharmaceutical Companies.
